# iBCE-EL: A New Ensemble Learning Framework for Improved Linear B-Cell Epitope Prediction

**DOI:** 10.3389/fimmu.2018.01695

**Published:** 2018-07-27

**Authors:** Balachandran Manavalan, Rajiv Gandhi Govindaraj, Tae Hwan Shin, Myeong Ok Kim, Gwang Lee

**Affiliations:** ^1^Department of Physiology, Ajou University School of Medicine, Suwon, South Korea; ^2^Department of Biological Sciences, Louisiana State University, Baton Rouge, LA, United States; ^3^Institute of Molecular Science and Technology, Ajou University, Suwon, South Korea; ^4^Division of Life Science and Applied Life Science (BK21 Plus), College of Natural Sciences, Gyeongsang National University, Jinju, South Korea

**Keywords:** B-cell epitope, ensemble learning, extremely randomized tree, gradient boosting, immunotherapy

## Abstract

Identification of B-cell epitopes (BCEs) is a fundamental step for epitope-based vaccine development, antibody production, and disease prevention and diagnosis. Due to the avalanche of protein sequence data discovered in postgenomic age, it is essential to develop an automated computational method to enable fast and accurate identification of novel BCEs within vast number of candidate proteins and peptides. Although several computational methods have been developed, their accuracy is unreliable. Thus, developing a reliable model with significant prediction improvements is highly desirable. In this study, we first constructed a non-redundant data set of 5,550 experimentally validated BCEs and 6,893 non-BCEs from the Immune Epitope Database. We then developed a novel ensemble learning framework for improved linear BCE predictor called iBCE-EL, a fusion of two independent predictors, namely, extremely randomized tree (ERT) and gradient boosting (GB) classifiers, which, respectively, uses a combination of physicochemical properties (PCP) and amino acid composition and a combination of dipeptide and PCP as input features. Cross-validation analysis on a benchmarking data set showed that iBCE-EL performed better than individual classifiers (ERT and GB), with a Matthews correlation coefficient (MCC) of 0.454. Furthermore, we evaluated the performance of iBCE-EL on the independent data set. Results show that iBCE-EL significantly outperformed the state-of-the-art method with an MCC of 0.463. To the best of our knowledge, iBCE-EL is the first ensemble method for linear BCEs prediction. iBCE-EL was implemented in a web-based platform, which is available at http://thegleelab.org/iBCE-EL. iBCE-EL contains two prediction modes. The first one identifying peptide sequences as BCEs or non-BCEs, while later one is aimed at providing users with the option of mining potential BCEs from protein sequences.

## Introduction

The humoral immune system is a complex network of cells that work together to protect the body against foreign substances or antigens such as bacteria, viruses, fungi, parasites, and cancerous cells. Generally, antigens are larger in size, however, only certain parts of antigenic determinants, called B-cell epitopes (BCEs), are recognized by specific receptors on the B-cell surface, generating soluble forms of antigen-specific antibodies (1). These antibodies play an important role in neutralization, cell-mediated cytotoxicity, and phagocytosis for the adaptive arm of immunity (2, 3). Thus, the identification and characterization of BCEs is a fundamental step in the development of vaccines, therapeutic antibodies, and other immunodiagnostic tools (4–7). Today, interest in epitope-based antibodies in biopharmaceutical research and development is rising due to their selectivity, biosafety, tolerability, and high efficacy.

B-cell epitopes are broadly classified into two categories: continuous/linear and discontinuous/conformational. Continuous/linear BCEs comprise linear stretches of residues in the antigen protein sequence, while the discontinuous/conformational BCEs comprise residues placed far apart in the antigen protein sequence, which are brought together in three-dimensional space through folding (8, 9). Experimental methods to identify BCEs include X-ray crystallography, cryo-EM, nuclear magnetic resonance, hydrogen–deuterium exchange coupled to mass spectroscopy, peptide-based approaches, mutagenesis, and antigen fragmentation (5, 10). However, these methods could be expensive and time-consuming. Therefore, new sequence-based computational methods need to be developed for rapid identification of potential BCEs. To this end, several computational methods based on machine learning (ML) algorithms have been developed to predict linear BCEs. These methods can be classified into local and global methods. Local methods such as Bcepred (11), BepiPred (12), and COBEpro (13) classify each residue as a BCE or non-BCE in a given protein sequence; global methods such as ABCpred (14), SVMTriP (15), IgPred (16), and LBtope (17) predict whether a given peptide is a BCE or non-BCE. Among global methods, LBtope is the most recently developed one and is also publicly available.

Although global prediction methods for linear BCEs have contributed to some development in this field, further studies are needed for the following reasons. (i) With the rapidly increasing number of BCEs in the Immune Epitope Database (IEDB) (18, 19), developing more accurate prediction methods using non-redundant (nr) benchmark data sets remain an important and urgent task. (ii) Most of the existing methods use random peptides as negative data sets. Experimentally determined negative data sets are necessary for developing efficient methods. Thus, better methods that use ML algorithms based on high-quality benchmarking data sets are necessary to accurately predict BCEs.

In this study, we constructed an nr data set of experimentally validated BCEs and non-BCEs from the IEDB and excluded sequences that showed more than 70% sequence similarity to avoid performance bias. We investigated six different ML algorithms [support vector machine (SVM), random forest (RF), extremely randomized tree (ERT), AdaBoost (AB), gradient boosting (GB), and *k*-nearest neighbors (*k*-NN)], five compositions [amino acid composition (AAC), amino acid index (AAI), dipeptide composition (DPC), chain-transition-distribution (CTD), and physicochemical properties (PCP)], 23 hybrid features (different combinations of the five compositions), and six binary profiles (BPF). We propose a novel ensemble approach, called iBCE-EL for predicting BCEs. The ensemble approach combines two different ML classifiers (ERT and GB) and uses the average predicted probabilities to make a final prediction. Furthermore, iBCE-EL achieved a significantly better overall performance on benchmarking and independent data sets and was capable of more accurate prediction than state-of-the-art predictor.

## Materials and Methods

### Construction of Benchmarking and Independent Data Sets

To build an ML model, an experimentally well-characterized data set is required. Therefore, we extracted a set of linear peptides from IEDB that tested positive for immune recognition (BCEs) and another set that tested negative (non-BCEs) (18, 19). Less than 1% of the peptides had lower than 5 or greater than 25 amino acid residues. We excluded these peptides from our data set because including them may result in outliers during prediction model development.

As mentioned in IEDB, one of the following seven different B-cell experimental assays (Qualitative binding, decreased disease, neutralization, disassociation constant KD, antibody-dependent cellular cytotoxicity, off rate, and on rate) are used to determine whether a peptide belongs to a positive or negative set of epitopes. Indeed, all this assay information is clearly specified for each peptide in IEDB (sixth column of the following link: http://www.iedb.org/bcelldetails_v3.php). It is worth mentioning that the criteria for categorizing positive and negative data set are the same as the one used in the recent study (12). To generate high confidence in our data set, we carefully examined each peptide assay information and considered as positive only when it has been confirmed as positive in two or more separate B-cell experiments. Similarly, peptides shown as negative in two or more separate experiment and never observed as positive in any of the above assays were considered as negative ones. To avoid potential bias and over-fitting in the prediction model development, sequence clustering and homology reduction using CD-HIT were performed, thus removing sequence redundancy from the retrieved data set. Based on the design of previous studies (20, 21), pairs of sequences that showed a sequence identity greater than 70% were excluded, thus obtaining an nr data set of 5,550 BCEs and 6,893 non-BCEs. Furthermore, each peptide present in our nr data set was mapped onto the original protein sequence, thus confirming the nature of linear epitopes. From this nr data set, 80% of the data was randomly selected as the benchmarking data set (4,440 BCEs and 5,485 non-BCEs) for development of a prediction model and the remaining 20% was used as the independent data set (1,110 BCEs and 1,408 non-BCEs).

### Feature Representation of Peptides

A peptide sequence (P) can be represented as:

(1) $$P=p_{1}p_{2}p_{3}\ldots p_{N}$$

where *p*_1_, *p*_2_, and *p*_3_, respectively, denotes the first, second, and third residues in the peptide *P*, and so forth. *N* denotes the peptide length. It should be noted that the residue *p_i_* is an element of the standard amino acid {A, C, D, E, F, G, H, I, K, L, M, N, P, Q, R, S, T, V, W, Y}. To train a ML model, we formulated diverse-length peptides as fixed-length feature vectors. We exploited five different compositions and BPF that cover different aspects of sequence information as described below:

(i) AAC
  Amino acid composition is the percentage of standard amino acids; it has a fixed length of 20 features. AAC can be formulated as follows:
  (2) $$AAC(P)=(f_{1},f_{2},f_{3},\ldots \ldots ,f_{20})$$
  where $f_{1}=\;\frac{R_{i}}{N}(i=1,2,\ldots ,20)$ is the percentage of composition of amino acid type *i, R_i_* is the number of type I appearing in the peptide, while *N* is the peptide length.
(ii) DPC
  Dipeptide composition is the rate of dipeptides normalized by all possible dipeptide combinations; it has a fixed length of 400 features. DPC can be formulated as follows:
  (3) $$DPC(P)=(f_{1},f_{2},f_{3},\ldots \ldots ,f_{400})$$
  where $f_{1}=\frac{R_{i}}{N}(i=1,2,\ldots ,400)$ is the percentage of composition of dipeptide type *i, R_i_* is the number of type *i* appearing in the peptide, while *N* is the peptide length.
(iii) CTD
  Chain-transition-distribution was introduced by Dubchak et al. (22) for predicting protein-folding classes. It has been widely applied in various classification problems. A detailed description of computing CTD features was presented in our previous study (23). Briefly, standard amino acids (20) are classified into three different groups: polar, neutral, and hydrophobic. Composition (C) consists of percentage composition values from these three groups for a target peptide. Transition (T) consists of percentage frequency of a polar followed by a neutral residue, or that of a neutral followed by a polar residue. This group may also contain a polar followed by a hydrophobic residue or a hydrophobic followed by a polar residue. Distribution (D) consists of five values for each of the three groups. It measures the percentage of the length of the target sequence within which 25, 50, 75, and 100% of the amino acids of a specific property are located. CTD generates 21 features for each PCP; hence, seven different PCPs (hydrophobicity, polarizability, normalized van der Waals volume, secondary structure, polarity, charge, and solvent accessibility) yields a total of 147 features.
(iv) AAI
  The AAindex database has a variety of physiochemical and biochemical properties of amino acids (24). However, utilizing all this information as input features for the ML algorithm may affect the model performance due to redundancy. Therefore, Saha et al. (25) classified these amino acid indices into eight clusters by fuzzy clustering method, and the central indices of each cluster were considered as high-quality amino acid indices. The accession numbers of the eight amino acid indices in the AAindex database are BLAM930101, BIOV880101, MAXF760101, TSAJ990101, NAKH920108, CEDJ970104, LIFS790101, and MIYS990104. These high-quality indices encode as 160-dimensional vectors from the target peptide sequence. Furthermore, the average of eight high-quality amino acid indices (i.e., a 20-dimensional vector) was used as an additional input feature. As our preliminary analysis indicated that both feature sets (160 and 20) produced similar results, we employed the 20-dimensional vector to save computational time.
(v) PCP
  Amino acids can be grouped based on their PCP, and this has been used to study protein sequence profiles, folding, and functions (26). The PCP computed from the target peptide sequence included (i) hydrophobic residues (i.e., F, I, W, L, V, M, Y, C, A), (ii) hydrophilic residues (i.e., S, Q, T, R, K, N, D, E), (iii) neutral residues (i.e., H,G, P); (iv) positively charged residues (i.e., K, H, R); (v) negatively charged residues (i.e., D, E), (vi) fraction of turn-forming residues [i.e., (N + G + P + S)/n, where *n* = sequence length], (vii) absolute charge per residue (i.e., $\left|\frac{R+K-D-E}{n}-0.03\right|$), (viii) molecular weight, and (ix) aliphatic index [i.e., (A + 2.9V + 3.9I + 3.9L)/*n*].
(vi) BPF

Each amino acid type of 20 different standard amino acids is encoded with the following feature vector 0/1. For instance, the first amino acid type A is encoded as b(A) = (1, 0, 0, …., 0), the second amino acid type C is encoded as b(C) = (0, 1, 0,…., 0), and so on. Subsequently, for a given peptide sequence P, its N or C-terminus with length of *k* amino acids was encoded as:

(4) $$BPF(k)=[b(p_{1}),b(p_{2}),\ldots ,b(p_{k})]$$

The dimension of BPF(*k*) is 20 × *k*. Here, we considered *k* = 5 and 10 both at N-terminus and C-terminus, which resulted BPFN5, BPFN10, BPFC5, and BPFC10. In addition to this, we also generated BPFN5-BPFC5 and BPFN10-BPFC10.

### Performance Assessment

A brief description of ML method employed in this study is given in the supplementary information, whose performances were evaluated using the receiver operating characteristic (ROC) analysis and the corresponding area under the ROC curve (AUC). An AUC value of 0.5 is equivalent to random prediction and an AUC value of 1 represents perfection. ROC analysis is based on the true positive rate and false positive rate at various thresholds. Furthermore, we used sensitivity, specificity, accuracy, and Matthews correlation coefficient (MCC) to assess prediction quality, which were defined as:

(5) $$\begin{array}{l} \text{Sensitivity}=\frac{\text{TP}}{\text{TP}+\text{FN}}\\ \text{Specificity}=\frac{\text{TN}}{\text{TN}+\text{FP}}\\ \text{Accuracy}=\frac{\text{TP}+\text{TN}}{\text{TP}+\text{TN}+\text{FP}+\text{FN}}\\ \text{MCC}=\frac{\text{TP}\times \text{TN-FP}\times \text{FN}}{\sqrt{\left(\text{TP}+\text{FP})(\text{TP}+\text{FN})(\text{TN}+\text{FP})(\text{TN}+\text{FN}\right)}} \end{array}$$

where TP is the number of true positives, i.e., BCEs classified correctly as BCEs, and TN is the number of true negatives, i.e., non-BCEs classified correctly as non-BCEs. FP is the number of false positives, i.e., BCEs classified incorrectly as non-BCEs, and FN is the number of false negatives, i.e., non-BCEs classified incorrectly as BCEs.

### Cross-Validation

In this study, we adopted the 5-fold cross-validation method, where benchmarking data set is randomly divided into five parts, from which four parts were used for training, and the fifth part was used for testing. This process was repeated until all the parts were used at least once as a test set, and the overall performance with all five parts was evaluated.

## Results

### Methodology Overview

**Figure 1:**
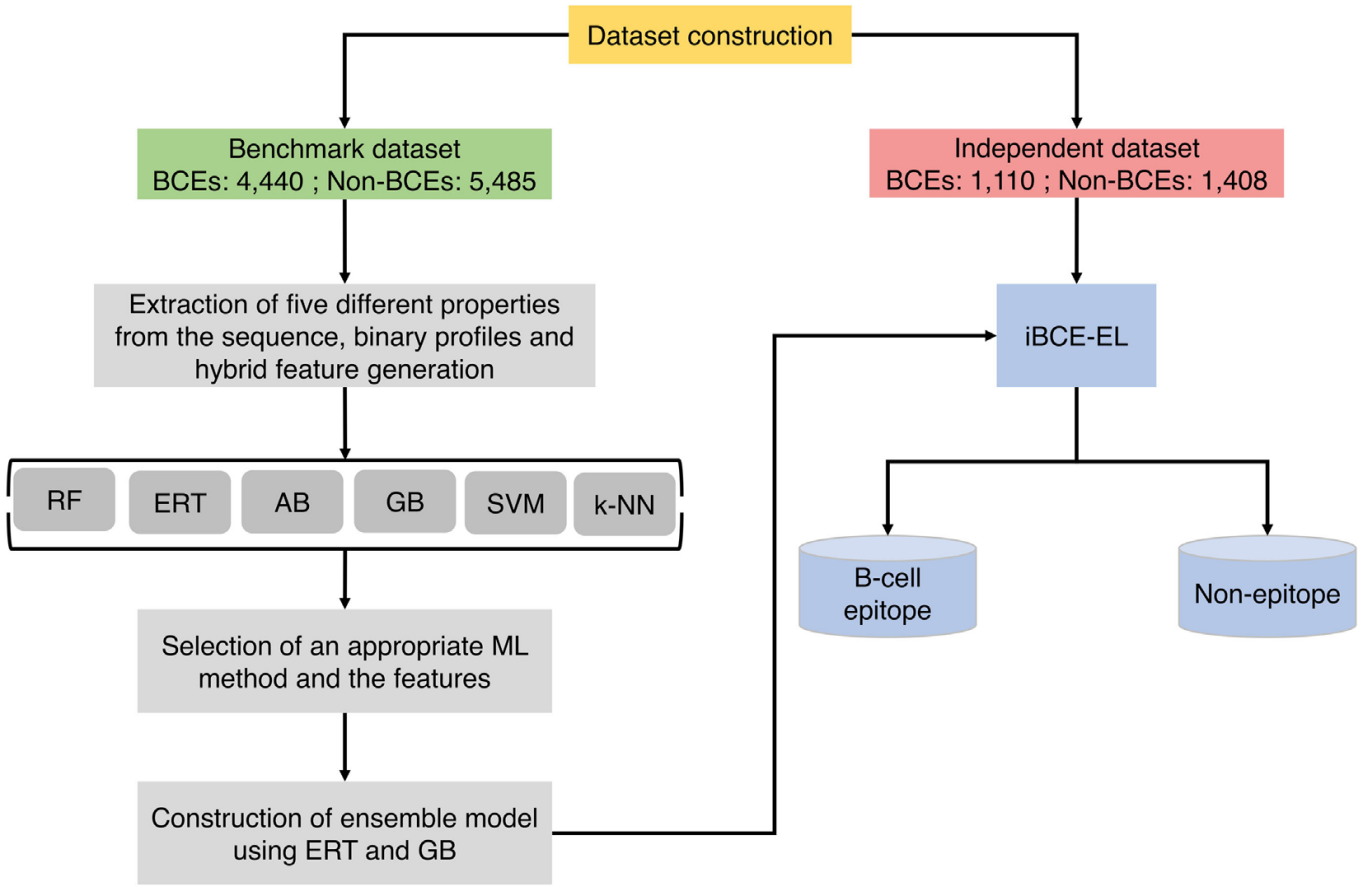
Overall framework of the proposed predictor. iBCE-EL development involved the following steps: (1) dataset curation, (2) feature extraction, (3) exploration of six different ML algorithms and selection of an appropriate algorithm and the corresponding features, and (4) construction of ensemble model.

Figure 1 shows a flowchart illustrating the methodology of iBCE-EL, which comprises four stages: (1) construction of an nr benchmarking data set of 9,925 peptides (4,440 BCEs and 5,485 non-BCEs) and an independent data set of 2,518 peptides (1,110 BCEs and 1,408 non-BCEs) from IEDB; (2) extraction of various features from peptide sequences, including AAC, AAI, CTD, DPC, and PCP, and generation of hybrid features (various combinations of individual compositions); (3) exploration of six different ML algorithms and selection of the appropriate ones and their corresponding features; and (4) construction of an ensemble model.

### Compositional and Positional Information Analysis

**Figure 2:**
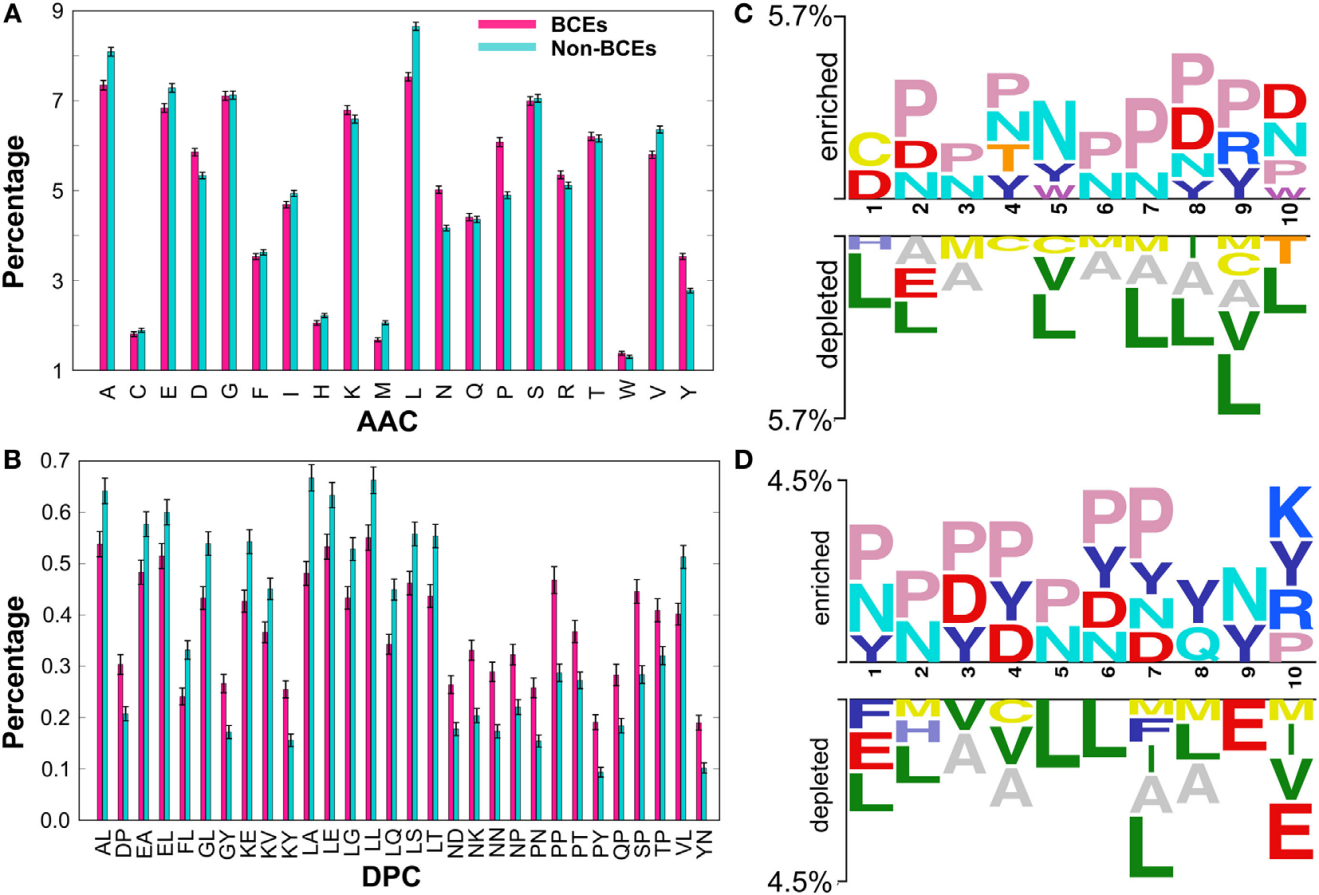
Compositional and positional preference analysis. **(A)** and **(B)** respectively represent the amino acid and dipeptide preferences of BCEs and non-BCEs. **(B)** Shows significant differences in top 30 dipeptides. **(C,D)** Represent positional conservation of 10 residues at the N- and C-terminals, respectively, between BCEs and non-BCEs, generated using two sample logos. In **(A,B)**, error bar is the SE that indicates the reliability of the mean. A smaller SE indicates that the sample mean is more accurate reflection of the actual population mean.

Prior to the development of the ML-based prediction model, we performed compositional analysis using combined data set (i.e., benchmarking and independent) to understand the nature of the preference of amino acid residues in BCEs and non-BCEs. AAC analysis showed that Asn (N), Asp (D), Pro (P), and Tyr (Y) were predominant in BCEs (Figure 2A). However, Ala (A), Glu (E), Leu (L), Val (V), and Met (M) were predominant in non-BCEs (Welch’s *t*-test; *P* ≤ 0.05). DPC analysis showed that 32.25% of dipeptides differed significantly between BCEs and non-BCEs (Welch’s *t*-test; *P* ≤ 0.05). Of these, the 10 most abundant dipeptides in BCEs and non-BCEs were PP, SP, NK, NN, PN, NP, KY, QP, PY, and DP and LA, LT, KE, LL, VL, LQ, GL, AL, LE, and LS, respectively (Figure 2B). These results suggested that the most abundant dipeptides in BCEs were mostly pairs of aromatic–aromatic residues or a positively or negatively charged residue paired with proline. The most abundant dipeptides in non-BCEs were aliphatic-aliphatic residues with hydroxyl group and aliphatic–aromatic amino acids. Overall, the differences observed in compositional analyses (AAC and DPC) can be used as an input feature for ML algorithms, where it can capture hidden relationships between features allowing a better classification. Therefore, we considered them as input features.

To better understand the positional information of each residue, sequence logos of the first 10 residues from the N- and C-terminals of BCEs and non-BCEs were generated using two sample logos (http://www.twosamplelogo.org). To test their statistical significance, the height of the peptide logos was scaled (*t*-test; *P* < 0.05). As shown in Figure 2C, at the N-terminal, Pro (P) at positions 2, 3, 4, and 6–10; Asn (N) at positions 2–8 and 10; Asp (D) at positions 1, 2, 8, and 10; and Tyr (Y) at positions 4, 5, 8, and 9 were significantly overrepresented, compared with other amino acids, while Leu (L) at positions 1, 2, 5, and 7–10; Ala (A) at positions 2, 3, and 6–9; Met (M) at positions 3, 6, 7, and 9; and Cys (C) at positions 4, 5, and 9 were significantly underrepresented. As shown in Figure 2D, at the C-terminal, Pro (P) at positions 1–7 and 10; Asn (N) at positions 1, 2, 5–7, and 9; Asp (D) at positions 3, 4, 6, and 7; and Tyr (Y) at positions 1, 3, 4, and 6–10 were significantly overrepresented, compared with other amino acids, while Leu (L) at positions 1, 2, and 5–8; Ala (A) at positions 3, 4, 7, and 8; Glu (E) at positions 1, 9, and 10; and Met (M) at positions 2, 7, 8, and 10 were significantly underrepresented. Notably, the predominant amino acids in the non-BCEs (particularly Leu, Val, and Met) were expected to be inside the proteins and if exist on the surface were likely to be present on the protein–protein interfaces. Conversely, the amino acids enriched in BCEs were mostly expected to be present on the protein surface. Overall, these results showed that BCEs and non-BCEs have contrasting amino acid preferences, which is consistent with the compositional analysis. Furthermore, positional preference analysis will be useful for researchers to design *de novo* BCEs by substituting amino acids at the specific position for increasing peptide efficacy. Interestingly, the properties of linear epitopes described here based on our data set are different from conformational epitopes (27), which is mainly due to the local arrangement of amino acids.

### Construction of Prediction Models Using Six Different ML Algorithms

**Figure 3:**
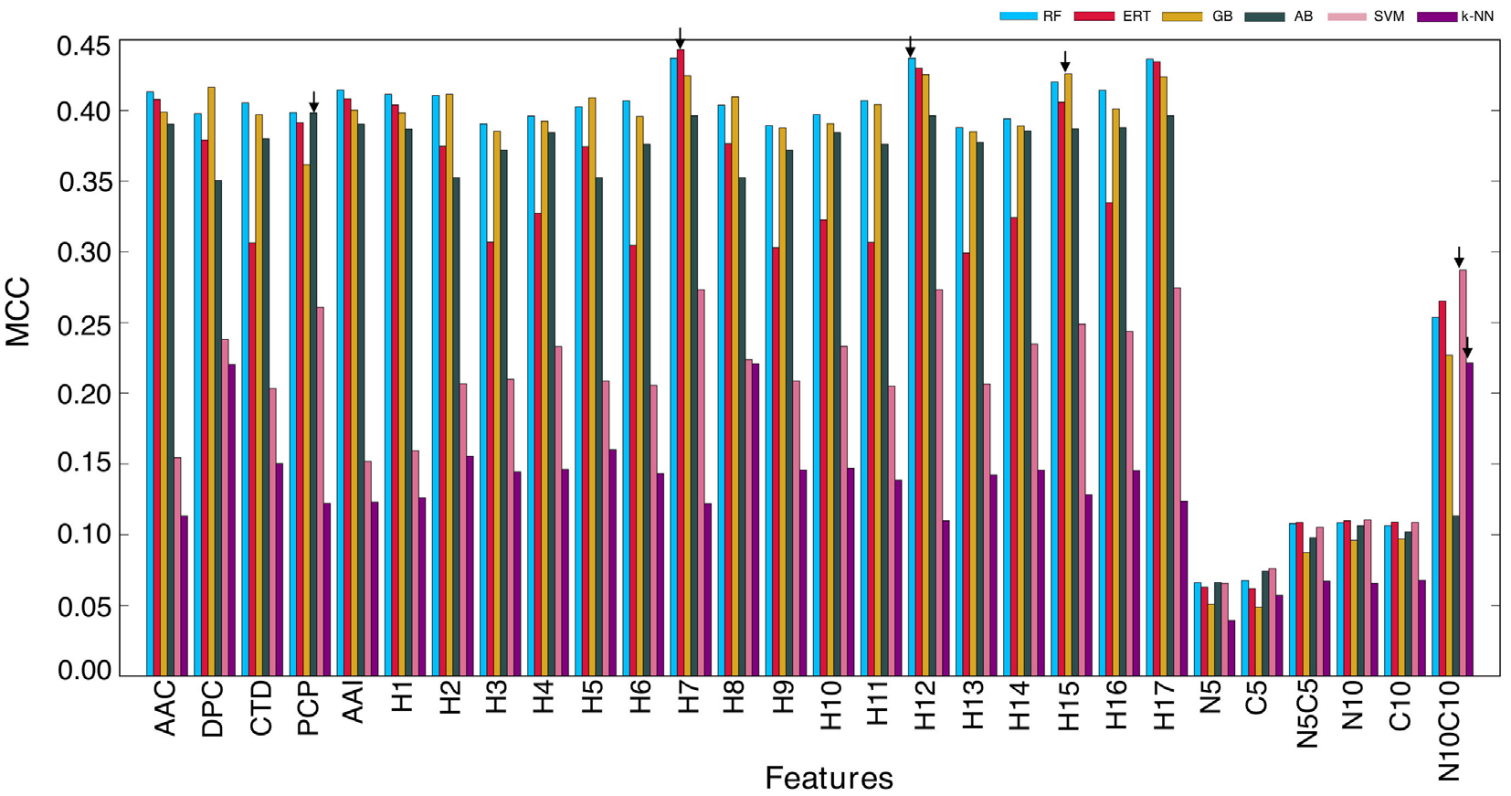
Performance of six different ML-based classifiers. Performance of various classifiers in distinguishing between B-cell epitopes (BCEs) and non-BCEs. A total of 27 classifiers were evaluated using 10 independent 5-fold cross-validation techniques, and their average performances in terms of AUC is shown. The final selected model for each ML-based method is shown with arrows. Abbreviations: AAC, amino acid composition; DPC, dipeptide composition; CTD, chain-transition-distribution; AAI, amino acid index; PCP, physicochemical properties; H1: AAC + AAI; H2: AAC + DPC + AAI; H3: AAC + DPC + AAI + CTD; H4: AAC + DPC + AAI + CTD + PCP; H5: AAC + DPC; H6: AAC + CTD; H7: AAC + PCP; H8: AAI + DPC; H9: AAI + DPC + CTD; H10: AAI + DPC + CTD + PCP; H11: AAI + CTD; H12: AAI + PCP; H13: DPC + CTD; H14: DPC + CTD + PCP; H15: DPC + PCP; H16: CTD + DPC; H17: AAC + AAI + PCP; N5: BPFN5; C5: BPFC5; N5C5: BPFN5 + BPFC5; N10: BPFN10; C10: BPFC10; and N10C10: BPFN10 + BPFC10.

In this study, we explored six different ML algorithms, including SVM, RF, ERT, GB, AB, and *k*-NN, using five different encoding schemes (AAC, AAI, CTD, DPC, and PCP) and their combinations (17 hybrid features), which included H1 (AAC + AAI); H2 (AAC + DPC + AAI); H3 (AAC + DPC + AAI + CTD); H4 (AAC + DPC + AAI + CTD + PCP); H5 (AAC + DPC); H6 (AAC + CTD); H7 (AAC + PCP); H8 (AAI + DPC); H9 (AAI + DPC + CTD); H10 (AAI + DPC + CTD + PCP); H11 (AAI + CTD); H12 (AAI + PCP); H13 (DPC + CTD); H14 (DPC + CTD + PCP); H15 (DPC + PCP); H16 (CTD + DPC); and H17 (AAC + AAI + PCP). Furthermore, we used six features set based on binary profiles, including BPFN5, BPFC5, BPFN5 + BPFC5, BPFN10, BPFC10, and BPFN10 + BPFC10. For each feature set, we used six different ML algorithms as inputs and optimized their corresponding ML parameters (Table S1 in Supplementary Material) using 5-fold cross-validation on the benchmarking data set. We repeated 5-fold cross-validation 10 times by randomly portioning the benchmarking data set and considering median ML parameters and average performance measures. The average performances of these six methods in terms of MCC is shown in Figure 3. RF, ERT, and GB performed consistently better than other ML-based methods (SVM, AB, and *k*-NN), regardless of the input features, indicating that decision tree-based methods are better suited for BCE prediction. Next, we investigated the features that produced the best performance for each ML algorithm. We found that SVM and *k*-NN performed best when using N10C10 binary profile as input feature; ERT, RF, GB, and AB performed best when H7, H12, H15, and PCP were used as input features, respectively. This analysis showed that the use of PCP-containing hybrid features as inputs could improve the performance of the ML method. Among the 6 ML methods, surprisingly, RF, ERT, and GB showed similar performances with MCC of 0.437, 0.443, and 0.426, respectively, which was significantly better than MCC of other 3 ML methods (SVM: 0.287, AB: 0.398, and *k*-NN: 0.221).

### Construction of iBCE-EL

**Figure 4:**
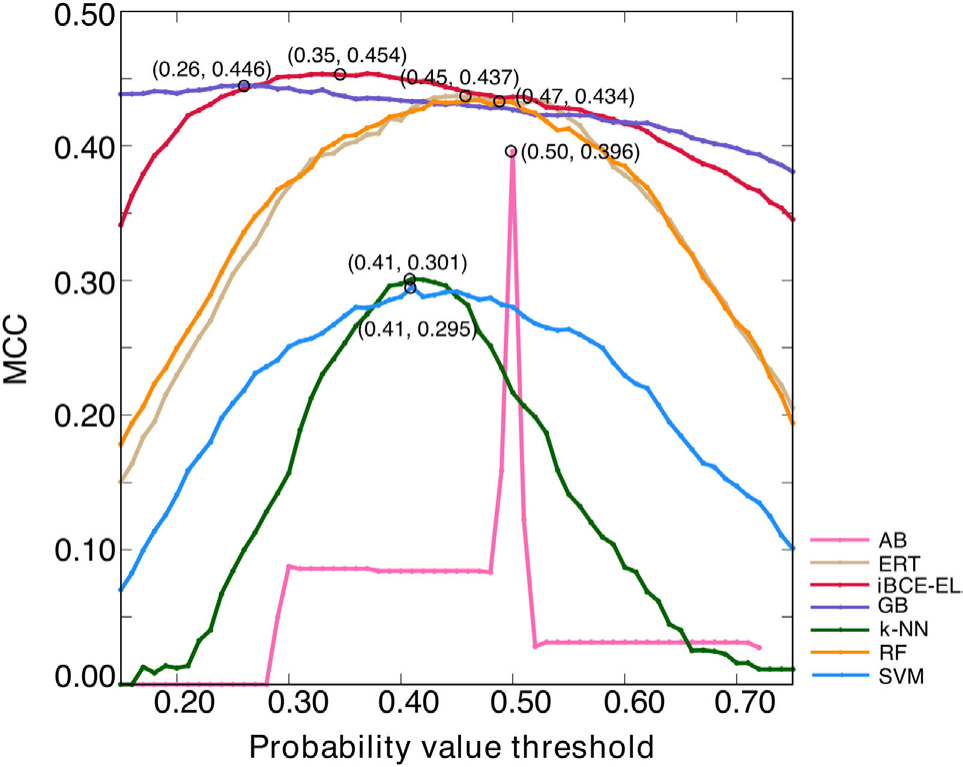
Optimization of probability value threshold. The *x*- and *y*-axes, respectively, represent the probability value threshold and Matthews correlation coefficient. The optimal value selected for each method is shown with a circle.

An ensemble model (EM) refers to a combination of several prediction models to make the final prediction (28). The major advantage of EMs over single models is the reported increase in robustness and accuracy (29). Here, we generated six ensemble models by combining different ML-based models, EM1 (GB + ERT); EM2 (GB + ERT + RF); EM3 (GB + ERT + RF + SVM); EM4 (GB + ERT + RF + SVM + AB); EM5 (GB + ERT + RF + SVM + AB + NN); and EM6 (GB + SVM + ERT). EM was calculated as follows: $\text{EM}=\frac{1}{n}\sum_{i=1}^{n}P_{i}$, where *n* is the number of ML-based models and *P* is the predicted probability value. Notably, we optimized the probability cut-off values (*P*) with respect to MCC using the grid search to define the class (BCEs or non-BCEs), which is a quite common approach and has been applied in various methods (30, 31). A model that produced the highest MCC was considered as the optimal one for each ensemble model. Surprisingly, all these ensemble models showed similar performances (Figure S1A in Supplementary Material) and hence it seems difficult to pick the best one. However, we checked its transferability on an independent data set and selected a model that showed consistent performance both on benchmarking and independent data sets (Figure S1B in Supplementary Material). According to this criterion, EM1 was selected as the best model and was labeled as iBCE-EL. To compare the performance of iBCE-EL with other ML-based models developed in this study, same optimization procedure was applied (Figure 4). Our results showed that iBCE-EL, RF, ERT, GB, AB, SVM, and *k*-NN produced the highest MCC with an optimal cut-off of 0.35, 0.47, 0.45, 0.26, 0.50, 0.41, and 0.41, respectively.

### Performance of Various Methods on Benchmarking Data Set

We compared the performance of iBCE-EL with that of the other 6 ML-based methods (RF, ERT, SVM, GB, AB, and *k*-NN). The results are shown in Table 1, where the methods are ranked according to the MCC associated with predictive capability. iBCE-EL had the highest MCC, accuracy, and AUC of 0.454, 0.729, and 0.782, respectively. Interestingly, MCC, accuracy, and AUC of iBCE-EL were 0.8–15.9, 0.4–9.5, and 0.6–21.9% higher than those of the other six ML-based methods (RF, ERT, SVM, GB, AB, and *k*-NN). McNemar’s Chi-square test (32) was used to evaluate the statistical significance of the differences in the performances of methods. At a *P*-value threshold of 0.05, iBCE-EL significantly outperformed SVM, *k*-NN, and AB and performed better than RF, ERT, and GB, thus indicating the superiority of iBCE-EL. To the best of our knowledge, iBCE-EL is the first ensemble approach for BCE prediction. For comparison, we also included LBtope (LBtope_variable_nr) cross-validation performance on an nr data set published previously (17). Although four variants are available for LBtope (LBtope_variable, LBtope_confirm, LBtope_variable_nr and LBtope_nr), LBtope_variable_nr is the only model that was developed using nr data set with variable length. Hence, we included only this model for comparison and evaluation. The accuracy, AUC, and MCC of iBCE-EL were higher than those of LBtope by ~6, 12.4, and 5.2%, respectively. To assess generalization and practical applicability of these models, we evaluated them using independent data set and compared their performances.

**Table 1:** Performance comparison of iBCE-EL with other ML-based methods on the benchmarking data set.

Method	Matthews correlation coefficient (MCC)	Accuracy	Sensitivity	Specificity	AUC	*P*-value
iBCE-EL	0.454	0.729	0.716	0.739	0.782	–
GB	0.446	0.725	0.712	0.735	0.773	0.051
ERT	0.437	0.718	0.734	0.705	0.776	0.205
RF	0.434	0.718	0.717	0.719	0.770	0.051
AB	0.396	0.702	0.662	0.722	0.737	**1.2E−16**
*k*-NN	0.301	0.644	0.715	0.591	0.691	**1.1E−9**
SVM	0.295	0.634	0.634	0.602	0.696	**<2.2E−16**
LBtope	0.330	0.667	0.660	0.672	0.730	–

*The first column represents the methods developed in this study. The columns 2–6 respectively represent the MCC, accuracy, sensitivity, specificity, and AUC value. The last column represents McNemar’s Chi-squared test was used to evaluate the performance between iBCE-EL and other methods. A *P* value <0.05 was considered to indicate a statistically significant difference between iBCE-EL and the selected method (shown in bold). For comparison, we have also included LBtope (LBtope_variable_nr) cross-validation performance on non-redundant data set*.

### Performance of Various Methods on Independent Data Set

By comparing the newly developed method with existing algorithms on the same data set, we could estimate the percentage of improvement. We compared the performance of iBCE-EL with those of LBtope and six other ML-based models. As shown in Table 2, iBCE-EL showed MCC, accuracy, and AUC of 0.463, 0.732, and 0.789, respectively. Indeed, the MCC, accuracy, and AUC of iBCE-EL were ~2.0–19.4, ~0.5–11.7, and ~1.0–10.4% higher than those of the other methods, thus indicating the superiority of iBCE-EL.

**Table 2:** Performance comparison of the iBCE-EL with other methods on independent data set.

Method	Matthews correlation coefficient (MCC)	Accuracy	Sensitivity	Specificity	AUC	*P*-value
iBCE-EL	0.463	0.732	0.742	0.724	0.789	–
GB	0.445	0.727	0.717	0.734	0.776	0.596
RF	0.434	0.718	0.718	0.718	0.777	0.839
ERT	0.440	0.719	0.742	0.703	0.780	0.476
AB	0.385	0.697	0.660	0.725	0.742	**2.4E−05**
LBtope	0.328	0.652	0.759	0.567	0.781	**7.4E−06**
*k*-NN	0.275	0.615	0.787	0.479	0.685	**4.9E−05**
SVM	0.269	0.624	0.721	0.548	0.694	**1.4E−05**

*The first column represents the method employed in this study. The columns 2–6, respectively, represent the MCC, accuracy, sensitivity, specificity, and AUC value. The last column represents McNemar’s Chi-squared test was used to evaluate the performance between iBCE-EL and other methods. A *P* value <0.05 was considered to indicate a statistically significant difference between iBCE-EL and the selected method (shown in bold). LBtope (LBtope_variable_nr) used SVM threshold of −0.1 to define the class as reported in Ref. (17)*.

**Figure 5:**
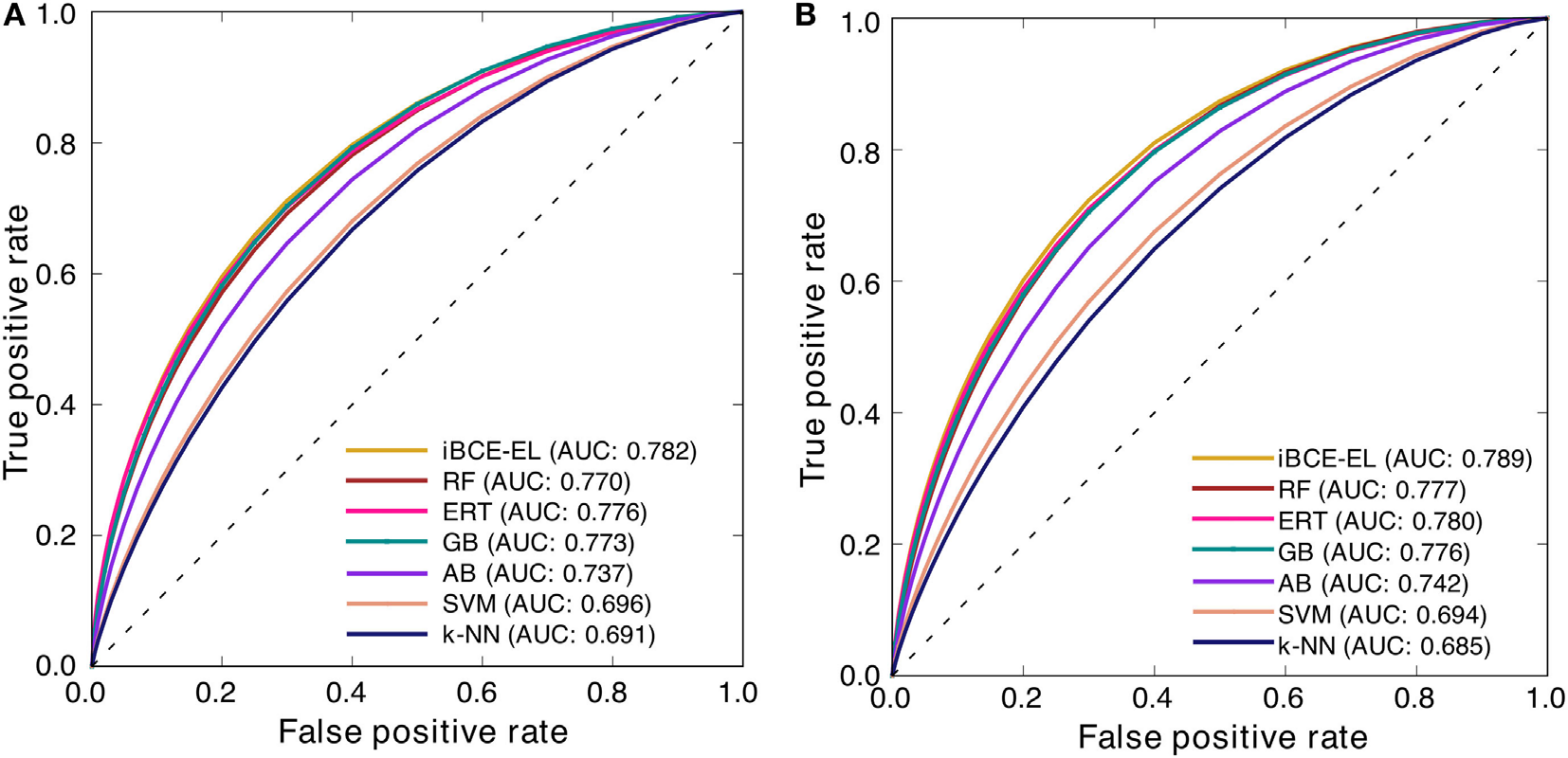
Receiver operating characteristic curves of the various prediction models. Results of 5-fold cross-validation on **(A)** a benchmarking data set and **(B)** independent data set.

At a *P*-value threshold of 0.05, iBCE-EL significantly outperformed SVM, AB, *k*-NN and LBtope, and performed better than ERT, RF and GB, thus indicating that our approach is indeed a significant improvement over the pioneering approaches in predicting linear BCEs. Interestingly, iBCE-EL performed consistently in both benchmarking and independent data sets (Figure 5) among the methods developed in this study suggesting its suitability for BCE prediction, despite the complexity of the problem. We made significant efforts to curate a large nr data set, explore various ML algorithms, and select an appropriate one for constructing an ensemble model thus resulting in consistent performance.

### Comparison of iBCE-EL With LBtope Methodology

We compared our method and LBtope (LBtope_variable_nr) in terms of algorithm characteristics. Since the variation in the number of B-cell experiments were considered to classify the peptides (positive or negative), LBtope used ~2-fold larger benchmarking data set than iBCE-EL. Moreover, we tested for significant differences in the data set using positional information analysis. However, we did not observe any significant differences between these two methods (Figure S2 in Supplementary Material). The choice of ML algorithm is different between these two methods, i.e., SVM used in LBtope, however, a combination of ERT and GB (ensemble model) were used in iBCE-EL. Interestingly, three features such as AAC, PCP, and DPC provide the most discriminative power for identifying BCEs; however, only DPC was used in LBtope.

### Web Server Implementation

Prediction methodologies available as web servers will be helpful for experimentalists, and several web servers for protein function predictions have been reported (23, 33–38). A web server has been developed to implement the iBCE-EL method and made publicly accessible at www.thegleelab.org/iBCE-EL for the use of the wider research community. Python, JAVA script, and HTML languages were employed to construct the web server. Users can submit amino acid sequences in the FASTA format. The output of the web server contains the class and predicted BCE probability values. The data set used in this study can also be downloaded from the iBCE-EL web server.

## Discussion

Computational identification of BCEs is one of the hot research topics in bioinformatics. An increasing number of experimentally validated BCEs is growing exponentially in IEDB, where most BCEs are found to be derived from protein sequences. To identify BCEs from a given protein sequence, experimental methods seem to be time-consuming, highly expensive, and complex to be utilized in a high-throughput manner. Therefore, recent efforts have focused on the development of computational methods to accelerate the identification of BCEs (12–15, 17, 39–46). Most existing BCE prediction methods were developed using very small data sets, with negative ones derived from randomly chosen peptides that are not experimentally validated (13–15, 17, 40, 42). This practice is quite common in other peptide-based prediction methods, including those for anticancer, antifungal, and cell-penetrating peptides (30, 47, 48). Among existing methods, LBtope is the latest publicly available tool with three different prediction models (17). It was developed using an nr data set that produced an accuracy of 66.7%, which is far from satisfactory. Hence, a novel method with better accuracy is necessitated. In this study, we developed a novel software called iBCE-EL, which allowed us to predict BCEs from a given primary peptide sequence based on the features derived from a set of experimentally validated BCEs and non-BCEs.

To the best of our knowledge, the data set we utilized was the most stringent redundancy-reduced data set with variable length of epitopes (12–25 amino acid residues). Recent studies demonstrated that BCEs with shorter lengths (7–12 amino acids) bind antibodies poorly (49). Therefore, such shorter peptides were not considered in our data set. In general, models developed using such high-quality data sets would have a wide range of applications in modern biology (50). Before developing the prediction model, we analyzed our data set to understand the compositional and positional preferences of BCEs and non-BCEs. We found that Pro and Asn were highly abundant in BCEs, compared to non-BCEs. These observations were consistent with the results of previous reports, where immunoglobulin binding antigenic regions were found to be rich in Pro/Gly (51, 52) residues. Future studies should focus on the experimental validation of the biological significance of various dipeptides we found to be involved in B-cell induction.

It is essential to explore different ML algorithms using the same data set and then select the best one, instead of arbitrarily selecting an ML algorithm (47, 53–58). We explored six different ML algorithms (SVM, RF, ERT, AB, GB, and *k*-NN) and 23 different features encoding schemes for classifying BCEs and non-BCEs. All the features and ML algorithms used in this study have been successfully applied in various sequence-based classification methods (53–55, 59–61); however, only SVM and DPC were used in LBtope (17). To the best of our knowledge, this is the first study to evaluate several ML algorithms for BCE prediction. Our systematic evaluation of features and ML algorithms revealed that RF, ERT, and GB showed similar performances, respectively, with a combination of PCP and AAI, a combination of PCP and AAC, and a combination of DPC and PCP as input features. Subsequently, we constructed an ensemble method called iBCE-EL by fusing ERT and GB. iBCE-EL performed better than individual component classifiers. The ensemble approach has been successfully applied for various problems, including signal peptide prediction (62), membrane protein type classification (63), protein subcellular location (64), and DNase I hypersensitive site prediction (65). However, this is the first instance where this approach has been utilized for BCE prediction. iBCE-EL performed significantly better than the existing method and six other methods developed in this study, when objectively evaluated on an independent data set. Interestingly, the performance of iBCE-EL was consistent on both benchmarking and independent data sets, thus indicating its ability to classify unseen peptides well when compared to other methods. The superior performance of iBCE-EL was primarily due to the larger size of the benchmarking data set, rigorous optimization procedures to select the final ML parameters, and the choice of ML methods to construct the ensemble model. Future studies should focus on identifying novel features that can be combined with the current feature set to further improve prediction performance. Furthermore, we expect that our proposed algorithm could also be applied to other fields of peptide or protein function prediction. Several authors still query whether BCE could be considered as a discrete feature of a protein molecule or not. Indeed, van Regenmortel suggests that an epitope is not an intrinsic feature of a protein molecule, but is a relational entity that can be defined only by its ability to react with the paratope of an antibody molecule (6, 27, 43, 49, 66).

In conclusion, we proposed a novel ensemble method called iBCE-EL to classify a given primary peptide sequence as BCE or non-BCE. The essential component of this study is the generation of high-quality data sets with several manually curated BCEs and non-BCEs. iBCE-EL showed consistent performance with both benchmarking and independent data sets, thus indicating its effectiveness and robustness. We have also created a user-friendly web interface, allowing researchers to use our prediction method. iBCE-EL is the second publicly available method for predicting BCEs, and its accuracy is remarkably higher than that of currently available methods. We anticipate that iBCE-EL will become a very useful tool for BCE prediction.

## Author Contributions

BM and GL conceived and designed the experiments. BM and RG performed the experiments. BM, RG, and TS analyzed the data. GL and MK contributed reagents/materials/software tools. BM, RG, and GL wrote the manuscript. All authors reviewed the manuscript and agreed to its submission in its present form.

## Conflict of Interest Statement

The authors declare that the research was conducted in the absence of any commercial or financial relationships that could be construed as a potential conflict of interest. The reviewer AM and handling Editor declared their shared affiliation.

## Supplementary Material

Figure S1 Optimization of probability value threshold. The *x*- and *y*-axes, respectively, represent the probability value threshold and MCC. The optimal value selected for each method is shown with a circle. **(A)** A benchmarking data set and **(B)** independent data set.

Figure S2 Comparison of position preference analysis using iBCE-EL and LBtope data set. **(A,B)** Represent positional conservation of 10 residues at N- and C-terminal, respectively, using iBCE-EL data set. **(C,D)** Represent positional conservation of 10 residues at N- and C-terminal, respectively, using LBtope data set.
