# Supplementary figures and images for: iBCE-EL: A New Ensemble Learning Framework for Improved Linear B-Cell Epitope Prediction
Source: Front Immunol. 2018 Jul 27;9:1695. doi: 10.3389/fimmu.2018.01695 (PMC6072840; doi:10.3389/fimmu.2018.01695)

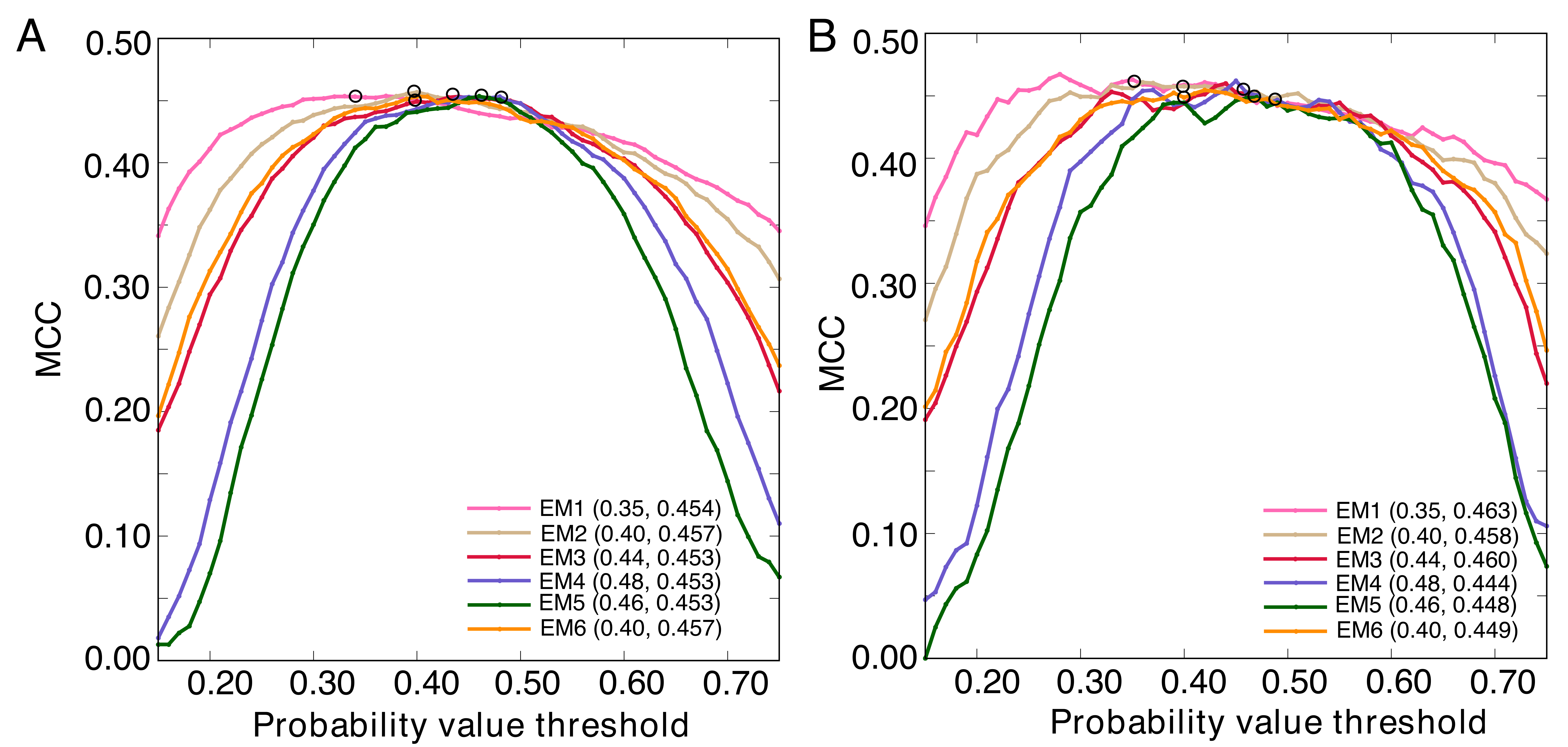

Supplement: Figure S1 — Optimization of probability value threshold. The x- and y-axes, respectively, represent the probability value threshold and MCC. The optimal value selected for each method is shown with a circle. (A) A benchmarking data set and (B) independent data set. [file image_1.tif]

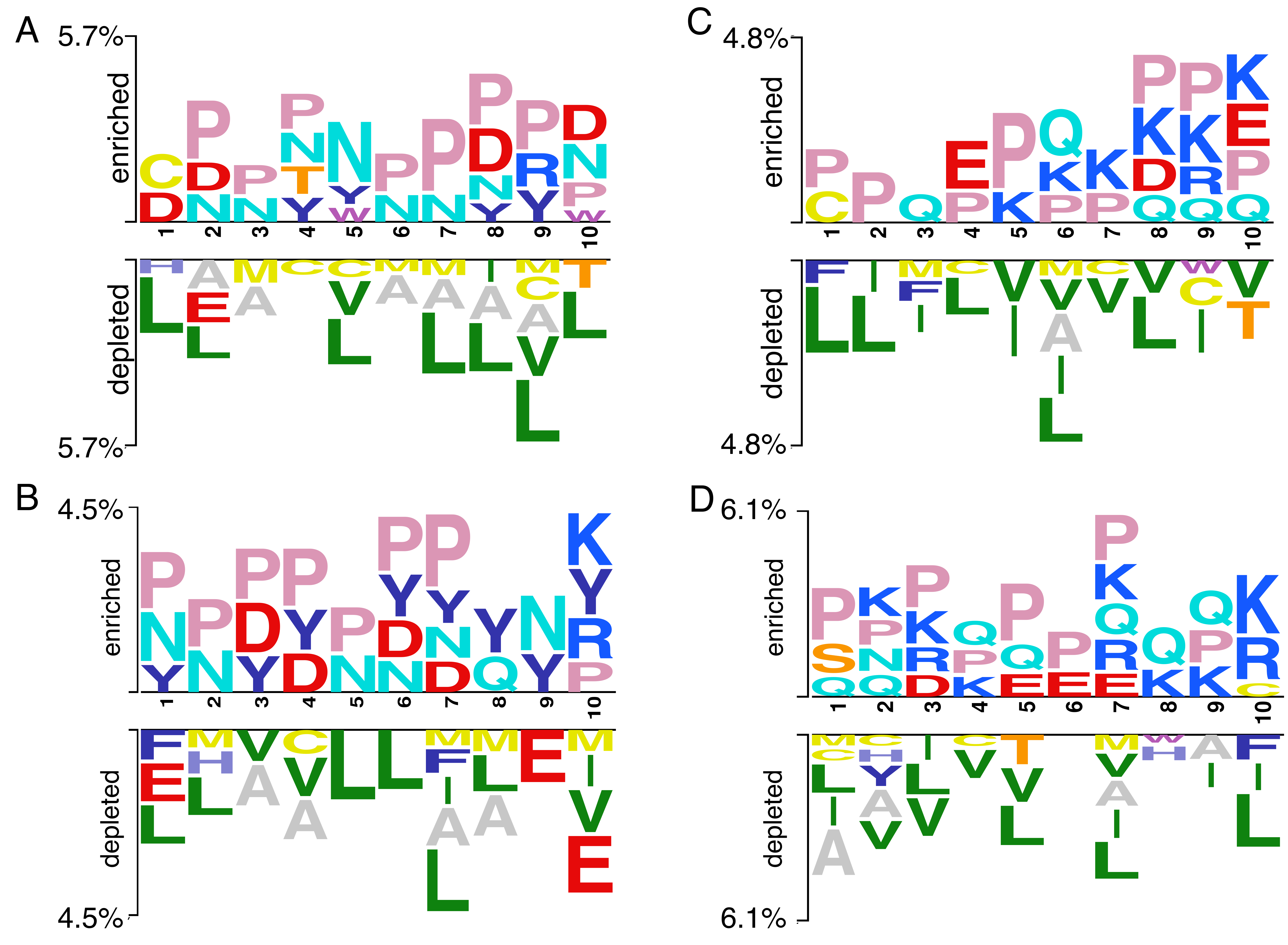

Supplement: Figure S2 — Comparison of position preference analysis using iBCE-EL and LBtope data set. (A,B) Represent positional conservation of 10 residues at N- and C-terminal, respectively, using iBCE-EL data set. (C,D) Represent positional conservation of 10 residues at N- and C-terminal, respectively, using LBtope data set. [file image_2.tif]
