# Supplementary material for: iBCE-EL: A New Ensemble Learning Framework for Improved Linear B-Cell Epitope Prediction
Source: Front Immunol. 2018 Jul 27;9:1695. doi: 10.3389/fimmu.2018.01695 (PMC6072840; doi:10.3389/fimmu.2018.01695)
Supplement: Supplementary file 3 [file data_sheet_1.docx]

**iBCE-EL: A New Ensemble Learning Framework for Improved Linear B-Cell Epitope Prediction**

***Balachandran Manavalan^1^, Rajiv Gandhi Govindaraj^2^, Tae Hwan Shin^1,3^, Myeong Ok Kim^4^ and Gwang Lee^1,3^****

*^1^ Department of Physiology, Ajou University School of Medicine, Suwon, Republic of Korea*

*^2^Department of Biological Sciences, Louisiana State University, Baton Rouge, LA, USA*

*^3^ Institute of Molecular Science and Technology, Ajou University, Suwon, Republic of Korea*

*^4^ Division of Life Science and Applied Life Science (BK21 Plus), College of Natural Sciences, Gyeongsang National University, Jinju, Republic of Korea*

Supplementary information

**Machine‐learning methods**

Due to the problem-specific nature of ML algorithms, we explored six different ML methods, including RF, AB, GB, *k*-NN, SVM, and ERT, which are suitable for binary classification (BCE and non-BCE). All these methods were implemented using the Scikit-Learn package (v0.18) [1]. A brief description of these methods and how they were used are presented in the following sections:

**(i) Support vector machine**

SVM performs both classification and regression problems [2], and has been successfully applied for protein function characterisation, protein functional site detection, transcription site prediction, and classification of gene expression data [3-8]. SVM transforms the input features into a high-dimensional feature space and determines the optimal separating hyperplane between two classes. A Gaussian radial-basis function (RBF) or Gaussian kernel was used to obtain the classification hyperplane. Two parameters required optimisation: *C* and *γ*; *C* is responsible for the trade-off between training error and margin, while *γ* defines how peaked Gaussians are centred on the support vectors. We employed a grid search in the range of 2^−15^ to 2^10^ for *C* and 2^−10^ to 2^10^ for *γ* (in log_2_-scale) to tune the SVM parameters (Table S1).

**(ii) Random forest**

RF utilises an ensemble of decision trees to perform classification and regression [9]; it has been successfully applied for various biological problems [10, 11]. This method has been described in detail in previous studies [12, 13]. In RF, the most influential tuning parameters are the number of trees (*ntree*), the number of variables randomly chosen at each node split (*mtry*), and the minimum number of samples required to split an internal node (*nsplit*).

**(iii) Extremely randomised trees**

ERT is another tree-based ensemble method, which constructs an ensemble of unpruned decision trees in a top-down manner [14]. Although ERT is similar to RF, the cut-points are randomly selected to divide nodes and the decision trees are constructed using the whole training dataset rather than the random subsets of training data. The parameters need to be optimised, similar to RF (Table S1).

**(iv) Gradient boosting**

Friedman, et al. [15] proposed a Gradient Tree Boosting (GB) algorithm, a forward learning ensemble method that performs both classification and regression problems. GB produces a prediction model in the form of an ensemble of weak prediction models, typically decision trees. GB consecutively fits new models to provide a more accurate estimate of the response variables, compared to other ensemble methods such as RF, ERT, and AB. The GB methodology has been described in detail in previous studies [16-19]. In GB, the three most influential parameters, *ntree*, *mtry*, and *nsplit*, require optimisation.

**(v) AdaBoost**

AdaBoost, also known as Adaptive Boosting, is an iterative algorithm that aims at achieving a weighted sum of boosted weak classifiers, especially decision trees. Previous studies have described this method in detail [20, 21]. In AB, *ntree* requires optimisation.

**(vi) *k*-Nearest neighbour**

*k*-NN is one of the simplest algorithms, and us used for both classification and regression. Here, we used the Euclidean distance to measure the distance function; the parameter *k* requires optimisation. Therefore, we optimised *k* using a grid search in the range of 1-300.

**References**

1. Pedregosa F, Varoquaux G, Gramfort A, Michel V, Thirion B, Grisel O, et al. Scikit-learn: Machine learning in Python. Journal of machine learning research. 2011;12(Oct):2825-30.

2. Chang C-C, Lin C-J. LIBSVM: a library for support vector machines. ACM transactions on intelligent systems and technology (TIST). 2011;2(3):27.

3. Yang ZR. Biological applications of support vector machines. Brief Bioinform. 2004;5(4):328-38. Epub 2004/12/21. PubMed PMID: 15606969.

4. Chen W, Yang H, Feng P, Ding H, Lin H. iDNA4mC: identifying DNA N4-methylcytosine sites based on nucleotide chemical properties. Bioinformatics. 2017;33(22):3518-23. Epub 2017/09/30. doi: 10.1093/bioinformatics/btx479. PubMed PMID: 28961687.

5. Manavalan B, Shin TH, Lee G. PVP-SVM: Sequence-Based Prediction of Phage Virion Proteins Using a Support Vector Machine. Front Microbiol. 2018;9:476. Epub 2018/04/05. doi: 10.3389/fmicb.2018.00476. PubMed PMID: 29616000; PubMed Central PMCID: PMCPMC5864850.

6. Manavalan B, Lee J. SVMQA: support-vector-machine-based protein single-model quality assessment. Bioinformatics. 2017;33(16):2496-503. Epub 2017/04/19. doi: 10.1093/bioinformatics/btx222. PubMed PMID: 28419290.

7. Yao B, Zhang L, Liang S, Zhang C. SVMTriP: a method to predict antigenic epitopes using support vector machine to integrate tri-peptide similarity and propensity. PLoS One. 2012;7(9):e45152. Epub 2012/09/18. doi: 10.1371/journal.pone.0045152. PubMed PMID: 22984622; PubMed Central PMCID: PMCPMC3440317.

8. Cao R, Wang Z, Wang Y, Cheng J. SMOQ: a tool for predicting the absolute residue-specific quality of a single protein model with support vector machines. BMC Bioinformatics. 2014;15:120. Epub 2014/04/30. doi: 10.1186/1471-2105-15-120. PubMed PMID: 24776231; PubMed Central PMCID: PMCPMC4013430.

9. Breiman L. Random forests. Machine learning. 2001;45(1):5-32.

10. Liu B, Long R, Chou KC. iDHS-EL: identifying DNase I hypersensitive sites by fusing three different modes of pseudo nucleotide composition into an ensemble learning framework. Bioinformatics. 2016;32(16):2411-8. Epub 2016/05/07. doi: 10.1093/bioinformatics/btw186. PubMed PMID: 27153623.

11. Gupta S, Mittal P, Madhu MK, Sharma VK. IL17eScan: A Tool for the Identification of Peptides Inducing IL-17 Response. Front Immunol. 2017;8:1430. Epub 2017/11/23. doi: 10.3389/fimmu.2017.01430. PubMed PMID: 29163505; PubMed Central PMCID: PMCPMC5671494.

12. Manavalan B, Shin TH, Kim MO, Lee G. AIPpred: Sequence-Based Prediction of Anti-inflammatory Peptides Using Random Forest. Frontiers in Pharmacology. 2018;9:276.

13. Manavalan B, Lee J, Lee J. Random forest-based protein model quality assessment (RFMQA) using structural features and potential energy terms. PLoS One. 2014;9(9):e106542. Epub 2014/09/16. doi: 10.1371/journal.pone.0106542. PubMed PMID: 25222008; PubMed Central PMCID: PMCPMC4164442.

14. Geurts P, Ernst D, Wehenkel L. Extremely randomized trees. Machine learning. 2006;63(1):3-42.

15. Friedman JH. Greedy function approximation: a gradient boosting machine. Annals of statistics. 2001:1189-232.

16. Ayaru L, Ypsilantis P-P, Nanapragasam A, Choi RC-H, Thillanathan A, Min-Ho L, et al. Prediction of outcome in acute lower gastrointestinal bleeding using gradient boosting. PloS one. 2015;10(7):e0132485.

17. Fan C, Liu D, Huang R, Chen Z, Deng L. PredRSA: a gradient boosted regression trees approach for predicting protein solvent accessibility. BMC Bioinformatics. 2016;17 Suppl 1:8. Epub 2016/01/29. doi: 10.1186/s12859-015-0851-2. PubMed PMID: 26818760; PubMed Central PMCID: PMCPMC4895273.

18. Pan Y, Liu D, Deng L. Accurate prediction of functional effects for variants by combining gradient tree boosting with optimal neighborhood properties. PLoS One. 2017;12(6):e0179314. Epub 2017/06/15. doi: 10.1371/journal.pone.0179314. PubMed PMID: 28614374; PubMed Central PMCID: PMCPMC5470696.

19. Tang Y, Liu D, Wang Z, Wen T, Deng L. A boosting approach for prediction of protein-RNA binding residues. BMC Bioinformatics. 2017;18(Suppl 13):465. Epub 2017/12/09. doi: 10.1186/s12859-017-1879-2. PubMed PMID: 29219069; PubMed Central PMCID: PMCPMC5773889.

20. Afolabi LT, Saeed F, Hashim H, Petinrin OO. Ensemble learning method for the prediction of new bioactive molecules. PLoS One. 2018;13(1):e0189538. Epub 2018/01/13. doi: 10.1371/journal.pone.0189538. PubMed PMID: 29329334; PubMed Central PMCID: PMCPMC5766097.

21. Raj A, Dewar M, Palacios G, Rabadan R, Wiggins CH. Identifying hosts of families of viruses: a machine learning approach. PLoS One. 2011;6(12):e27631. Epub 2011/12/17. doi: 10.1371/journal.pone.0027631. PubMed PMID: 22174744; PubMed Central PMCID: PMCPMC3235098.
