# Supplementary material for: iBCE-EL: A New Ensemble Learning Framework for Improved Linear B-Cell Epitope Prediction
Source: Front Immunol. 2018 Jul 27;9:1695. doi: 10.3389/fimmu.2018.01695 (PMC6072840; doi:10.3389/fimmu.2018.01695)
Supplement: Supplementary file 4 [file table_1.docx]

**Table S1.** Hyperparameters optimised by grid search

| **Method** | **Parameter** | **Tested values** |
| --- | --- | --- |
| RF, ERT, and GB | Number of estimators (n_estimators^[a]^) | 60–1000 with an interval of 20 |
|  | Maximum number of features considered per split (max_features^[a]^) | 1–20 with an interval of 1, “sqrt^[b]^”, None^[c]^ |
|  | Minimum number of samples required to split an internal node (min_samples_split^[a]^) | 2–10 with an interval of 1 |
| SVM | Penalty parameter (C^[a]^) | [2^-15^–2^10^] in log_2_ steps |
|  | Kernel coefficient ($\gamma$^[a]^) | [2^-10^–2^10^] in log_2_ steps |
| AB | Maximum number of estimators at which boosting is terminated (n_estimator^[a]^) | 20-1000 with an interval of 20. |
| *k*-NN | Number of neighbors (n_neighbors^[a]^) | 1-150 with an interval of 1. |

Column 1 represents the ML algorithms. Column 2 and 3 respectively represent the parameter and the tested values. ^[a]^ Parameter name in the scikit-learn implementation. ^[b]^ default value. ^[c]^ all features are used.
